# Evaluation of Event-Free Survival Surrogating Overall Survival as the Endpoint in Neoadjuvant Clinical Trials of Gastroesophageal Adenocarcinoma

**DOI:** 10.3389/fonc.2022.835389

**Published:** 2022-04-07

**Authors:** Hua Liu, Yakun Wang, Changsong Qi, Tong Xie, Zhi Peng, Jian Li, Lin Shen, Xiaotian Zhang

**Affiliations:** Department of Gastrointestinal Oncology, Key Laboratory of Carcinogenesis and Translational Research (Ministry of Education), Peking University Cancer Hospital & Institute, Beijing, China

**Keywords:** gastric and gastroesophageal junction adenocarcinoma, neoadjuvant therapy, overall survival, event-free survival, surrogate endpoint

## Abstract

**Background:**

Gastric cancer (GC) is one of the most common malignant cancers worldwide. The development of potential antitumor agents is being investigated and stimulates more clinical trials. Overall survival (OS) is consistently considered the primary endpoint for clinical trials on treatment effect assessment. However, finding an appropriate endpoint more sensitive and easy for trials is vital. For adjuvant chemotherapy, current evidence has shown that disease-free survival (DFS) could be a surrogate endpoint for randomized controlled trials (RCTs) with GC, but evidence for neoadjuvant chemotherapy (NCT) or chemoradiotherapy (NCRT) is inadequate. This study was designed to evaluate the possibility that event-free survival (EFS) surrogates OS in RCTs of NCT/NCRT of gastric orss gastroesophageal (GC or GEJ) adenocarcinoma patients (ADK).

**Methods:**

A literature search was conducted through databases including PubMed, the Cochrane Library, and Embase. References and articles from other sources were also included. A total of 8 RCTs with 2,837 patients were eventually analyzed. Hazard ratios (HRs) of OS and EFS were directly approached. The surrogacy of EFS was assessed through the correlation of determination R^2^. We used Review Manage pooling HRs of OS and EFS at the trial level. I^2^ was used to demonstrate the heterogeneity of inclusions. Publication bias was summarized and illustrated through funnel plots. All analyses were on two sides with a setting statistical significance as p < 0.05.

**Results:**

Eight RCTs of 2,837 patients were analyzed at the trial level. The I^2^ for OS was 21% and 51% for EFS, and a fixed-effect model was used. The pooled HR of OS was 0.83 (95% CI: 0.75–0.92, p < 0.001), and that of EFS was 0.78 (95% CI: 0.71–0.86, p < 0.001). The regression correlation coefficient between EFS and OS was 0.76 (95% CI: 0.41–1.11, p = 0.002), and the coefficient of determination R^2^ = 0.826.

**Conclusions:**

A strong correlation was observed between OS and EFS at the trial level. EFS could be a surrogate endpoint for neoadjuvant RCTs of GC and GEJ adenocarcinoma. Further studies and evidence from individual data are expected.

## Introduction

With more than a million new diagnoses per year, gastric cancer (GC) ranks as one of the most common malignant cancers worldwide. It remains the fourth main cause of cancer-related deaths (1). Among all types of histology, gastric and gastroesophageal adenocarcinoma (GC and GEJ ADK) is the most common histologic type of GC (2). Most patients found themselves later staged after diagnosis. Therapies and maintenance of the quality of life could be an enormous economic and health load for people and society, although in general, the past 5 years has seen a decline in both GC incidence and mortality. Although cancer screening at the early phase is an effective tool to discover GC in the early stage and provide chances for endoscopic resection, it is still difficult and lacks conduction nationwide, and a considerably large proportion of GC was found to be advanced.

Surgery is the main and curative strategy for resectable GC or GEJ ADK, but the effect of surgery alone is inadequate, especially for locally advanced GC (3). For late, widespread, or locally advanced patients, in addition to surgical operations, chemotherapy or radiochemotherapy also plays an important role in cancer treatment strategies and patient long-term survival. An increasing number of studies have been developed to explore better combinations or sequential schemes. Perioperative chemotherapy was gradually approved before and after surgery from a series of randomized controlled trials (RCTs). Neoadjuvant chemotherapy (NCT) before surgery could downstage or control micrometastasis of the resectable tumor before surgery or offer surgery chances to patients with unresectable tumors. For survival, the practice of NCT and surgery might have a positive influence on the outcome of GC or GEJ ADK patients (4). New and potential antitumor agents of various pathways and targets have been searched, and more clinical trials with appropriate and more sensitive endpoints have been stimulated to validate the effect of new treatment strategies (5).

Overall survival (OS) is commonly accepted as the gold standard endpoint in these trials. This means that the time from randomization to death of any reason or the last follow-up data is censored (6, 7). It is easy to measure and interpret and can show clinical benefit directly. Nevertheless, OS requires a long follow-up period and could be easily influenced by subsequent treatments. Additionally, new therapies usually face potentially limited benefits and need large samples to demonstrate them. OS is a comparatively rough endpoint for precise effect variation; therefore, more sensitive endpoints are expected, such as disease-free survival (DFS), progression-free survival (PFS), and event-free survival (EFS). The definition of DFS is the time from randomization to any disease recurrence in the local region, distant metastases or second primary cancer, or death from any cause (6, 7). EFS is defined as the time from randomization until the progression of diseases precluding surgery, local or distant recurrence, or death of any cause (8). PFS is defined as the time during randomization and objective tumor progression or death, which occurs first. EFS and PFS could be considered the same endpoints for neoadjuvant clinical trials, while the same consideration of DFS should be more cautious, which usually is calculated after surgery. The actual application of the definition of DFS and EFS is intricate and considers deaths of all causes as recurrence can minimize analysis bias (6). In some situations with prolonged survival, OS could be impractical, and EFS is an important endpoint. The follow-up time needed for EFS is much shorter than that needed for OS, and it could be a more appropriate endpoint for covering clinical profit when the quality of life and adverse events of agents are considered. It could not be influenced by subsequent treaments. This article aims to evaluate the probability of EFS as a suitable surrogate endpoint of OS in NCT or neoadjuvant chemoradiotherapy (NCRT) RCTs of GC and GEJ ADK.

## Methods

### Trial Search and Study Selection

Studies were searched through PubMed, the Cochrane Library, and Embase, and the references cited by the included studies, relative reviews, and meta-analyses were also searched. The whole search procedure lasted from January 10, 2021, to February 10, 2021. The literature search strategy in PubMed was (((((gastric cancer) OR (gastric adenocarcinoma)) OR (gastric neoplasms)) OR (gastroesophageal junction adenocarcinoma)) AND ((((neoadjuvant chemotherapy) OR (neoadjuvant therapy)) OR (neoadjuvant chemoradiotherapy))) OR (neoadjuvant drugs))) AND ((((overall survival) OR (disease-free survival)) OR (event-free survival)) OR (progression-free survival)). The keywords were consistent in other databases. Unpublished studies were not searched, and a recently published study was taken into the final analysis. Articles meeting the following criteria were included: 1) enrolling patients with GC or GEJ ADK; 2) comparing the effect of NCT and surgery alone, or NCRT and NCT between two arms; 3) RCT; 4) OS and EFS or DFS or PFS being used as endpoints for study; and 5) hazard ratios (HRs) of endpoints for two comparative arms could be reached. Exclusion criteria consist of the following: 1) patients with other tumor histology or location except for GC or GEJ ADK; 2) no neoadjuvant therapy related or other topics not comparing treatment effect of neoadjuvant therapy; 3) non-English publication; 4) articles of systematic reviews, case reports, replies, and meta-analyses; 5) studies of non-RCTs; 6) no OS or EFS or DFS or PFS data available; 7) HR could not be accessed directly from the study; 8) abstracts, articles of trial design, or conference summary; or 9) no full-text available studies. All titles and abstracts of the searched results were first scanned, and then the full text was carefully browsed. The study selection was conducted separately by two researchers on our study team. When there were different opinions about study selection, the discussion was conducted, and agreement eventually was reached.

### Data Extraction and Study Quality Assessment

Countries, number of patients, tumor location, tumor histology, therapy regimen, and other basic characteristics of the experimental and control arms were extracted. HRs published were directly reached and used at the trial level. The Cochrane risk-of-bias tool for RCTs was used to assess the quality of studies. The low, high, or unclear risk was determined for each included trial.

### Statistical Analysis

We used Review Manager (RevMan, version 5.3, the Nordic Cochrane Centre, the Cochrane Collaboration, Copenhagen, Denmark) to pool trial-level HRs of OS and EFS, and a 95% CI was applied. I^2^ was used to demonstrate the heterogeneity of studies. Funnel plots were used to evaluate publication bias, and no publication bias was set as symmetric funnel plots within the 95% region. The strength of the correlation between the HRs of OS and EFS was evaluated by linear regression using SPSS (version 26.0; SPSS Inc., Chicago, IL, USA). All analyses were on two sides with a setting statistical significance as p < 0.05.

## Results

### Literature Search and Quality Assessment

**Figure 1:**
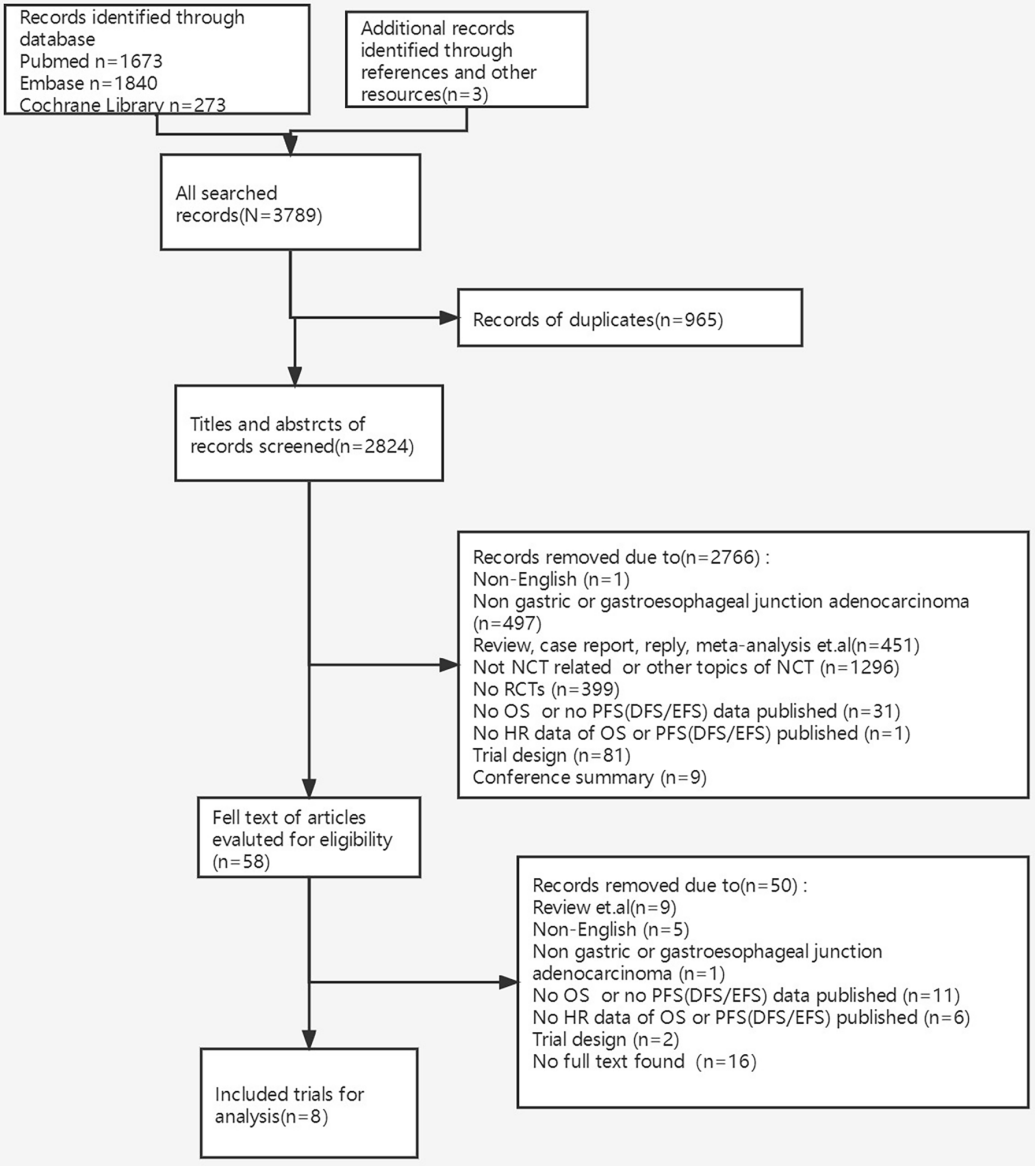
The procedure of identification, screening, eligibility, and inclusion of trials. NCT, neoadjuvant chemotherapy; RCTs, randomized controlled trials; OS, overall survival; PFS, progression-free survival; DFS, disease-free survival; EFS, event-free survival; HR, hazard ratio.

In total, 3,789 studies were initially identified, and 8 RCTs of 2,837 patients were eventually included for final analysis after screening titles, abstracts, and then full texts (9–11) (12–14) (15, 16). Detailed information on the procedure is shown in **Figure 1**. All included RCTs were at low risk of bias taking random sequence generation, allocation concealment, incomplete outcome data, and selective reporting into account.

### Characteristics and Demographics of Inclusions

The basic characteristics of the trials are shown in **Table 1**. Eight trials were published from 2006 to 2021, and the patient enrollment process ranged from 1994 to 2017 (**Table 1**). All included studies were phase III RCTs, and 6 trials were conducted in European countries. Only Iwasaki et al. (14) and Kang et al. (16) were separately conducted in Japan and Korea. Except for Cunningham et al. (10) and Ychou et al. (11), who included patients with lower third esophageal cancer, the study population all had GC or GEJ. The average age of the study population was 56 to 64 years old; most were male and had an Eastern Cooperative Oncology Group (ECOG) status of 0. OS was set as the primary endpoint.

**Table 1:** Basic characteristics of eligible trials.

Trial	Country	Trial phase	Tumor location/tumor histology	Follow time: exp/ctr (months)	HR_OS_ (95% CI)	HR_EFS_ (95% CI)	Ctr. arm		Exp. arm		Another endpoint	Clinical stage
							Therapy	Number of patients	Therapy	Number of patients		
Schuhmacher 2010	Europe	III	GC, GEJ; ADK	56.4/53.3	0.84 (0.52–1.35)	0.76 (0.49–1.16)	S	72	CT→S	72	PFS	III–IV (the 5th UICC)
Cunningham 2006	Europe	III	GC, GEJ, lower third esophageal; ADK	49/47	0.75 (0.60–0.93)	0.66 (0.53–0.81)	S	253	CT→S→CT	250	PFS	–
Ychou 2011	French	III	GC, GJ, lower third esophageal; ADK	68.4	0.69 (0.50–0.95)	0.65 (0.48–0.89)	S	111	CT→S	113	DFS	–
Shapiro 2015^‡^	Netherlands	III	GS,GJ, esophageal^‡^; ADK	84.1^‡^	0.73 (0.55–0.98)	0.69 (0.52–0.92)	S	141	CRT→S	134	PFS	–
Stahl 2017	Europe	III	GEJ; ADK	126.5/126.5	0.65 (0.42–1.01)	0.64 (0.39–1.06)	CT→S	59	CRT→S	60	PFS	III–IV (the 5th UICC)
Iwasaki 2020	Japan	III	GC; ADK	54	0.92 (0.68–1.24)	0.98 (0.74–1.29)	S→CT	149	CT→S→CT	151	PFS	IB-IV (the JCGC, 2nd English version)
Cats 2018	Europe	III	GC, GEJ; ADK	61.4	1.01 (0.84–1.22)	0.99 (0.82–1.19)	CT→S→CT	393	CT→S→CRT	395	EFS	IB-IVA (the 6th AJCC)
Yoon-Koo Kang 2021	Korea	III	GC, GEJ; ADK	38.6	0.84 (0.60–1.19)	0.70 (0.52–0.95)	S→CT	246	CT→S→CT	238	PFS	IIA–IIIC (the 7th AJCC)

GC, gastric cancer; GEJ, gastroesophageal junction; ADK, adenocarcinoma; CT, chemotherapy; CRT, chemoradiotherapy; Ctr. arm, control arm; Exp. arm, experiment arm; PFS, progression-free survival; DFS, disease-free survival; EFS, event-free survival; JCGC, the Japanese Classification of Gastric Carcinoma; UICC, the Union for International Cancer Control; AJCC, the American Joint Committee on Cancer; HR, hazard ratio.

^‡^Demographic characteristic data included the population of esophageal squamous cell carcinoma, and HRs of adenocarcinoma were only extracted and taken into analysis.

The clinical stage in the two studies was III-IV *via* the 5th Union for International Cancer Control (UICC), IB-IVA in one study *via* the 6th American Joint Committee on Cancer (AJCC), IIB-IV in one study *via* the 7th AJCC, and IB-IV in one study through the Japanese Classification of Gastric Carcinoma, 2nd English version. The clinical stage in the other three articles was unavailable (**Table 1**).

Six studies utilized PFS as the other endpoint. Cats et al. (15) used EFS and Ychou et al. (11) used DFS, which in the article was “calculated from a landmark time of 6 months after date of random assignment to allow the difference in the timing of surgery between the two treatment groups and a modification of the logrank procedure was used. Events, including incomplete resection, local and distant recurrence, and death, arising within the first 6 months were regarded as events at this landmark time” [(Ychou et al. (11)]. Subsequently, although different second endpoints were used in the studies, the definition of DFS, EFS, and PFS was coincidently identified, and it was reasonable to consider DFS, EFS, and PFS as the same endpoint in our study. It was defined as the time from randomization to local or distant recurrence or disease progression or unresectable disease before surgery or death of any cause.

### Pooled Overall Survival and Event-Free Survival Hazard Ratio of Inclusions

**Figure 2:**
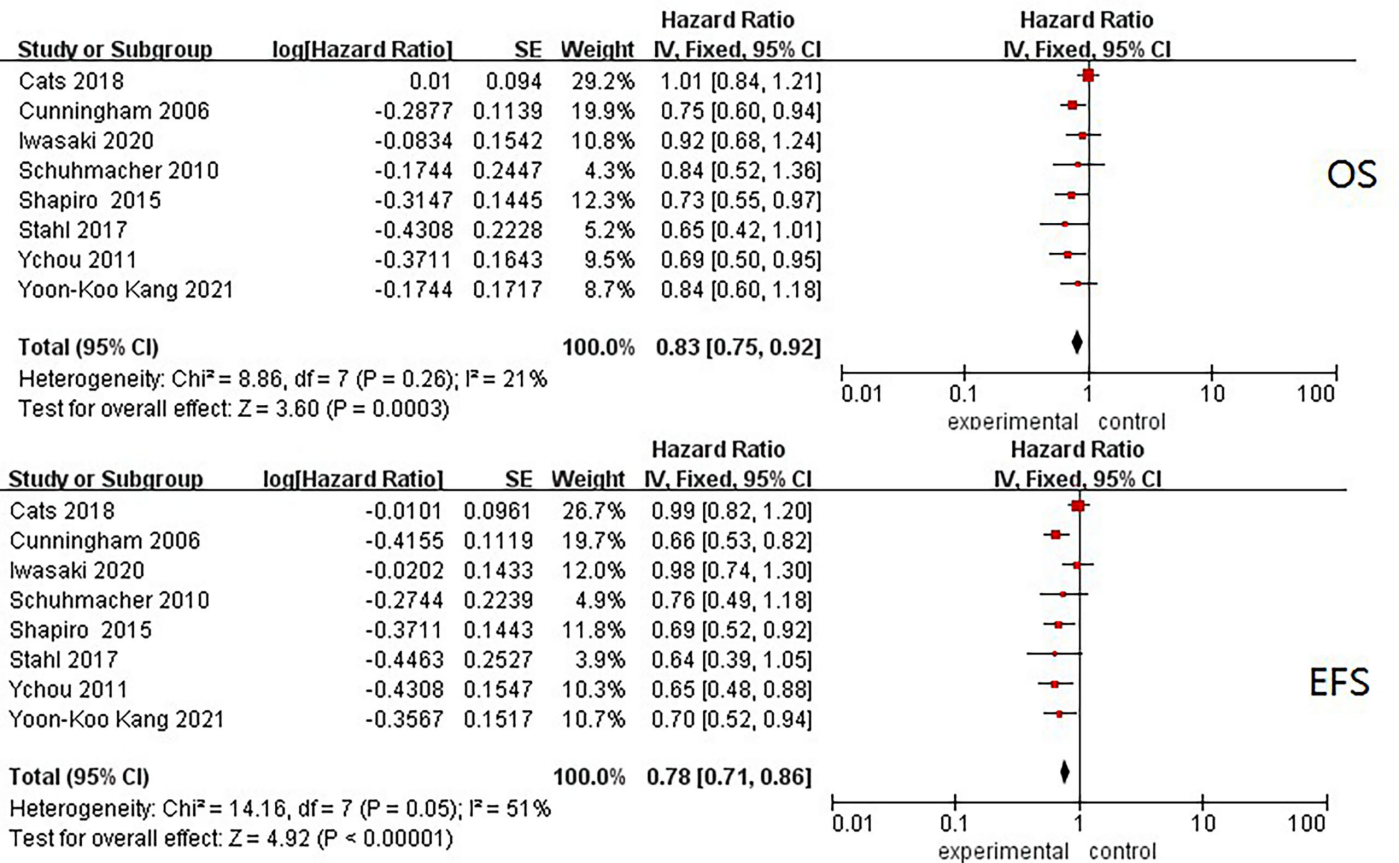
Forest plots of HRs of EFS and OS from all included neoadjuvant trials. OS, overall survival; EFS, event-free survival; HR, hazard ratio.

In total, trial-level data of 2,837 patients were put into the analysis, and pooled HRs of OS and EFS were separately obtained. For OS, the I^2^ was 21%, and the pooled HR was 0.83 (95% CI: 0.75–0.92, p < 0.001) in a fixed-effect model. The I^2^ of EFS was 51%, and the pooled HR was 0.78 (95% CI: 0.71–0.86, p < 0.001). Similar pooled HRs were reached for OS and EFS, with low–middle heterogeneity shown among the included studies (**Figure 2**).

### Trial-Level Association

**Figure 3:**
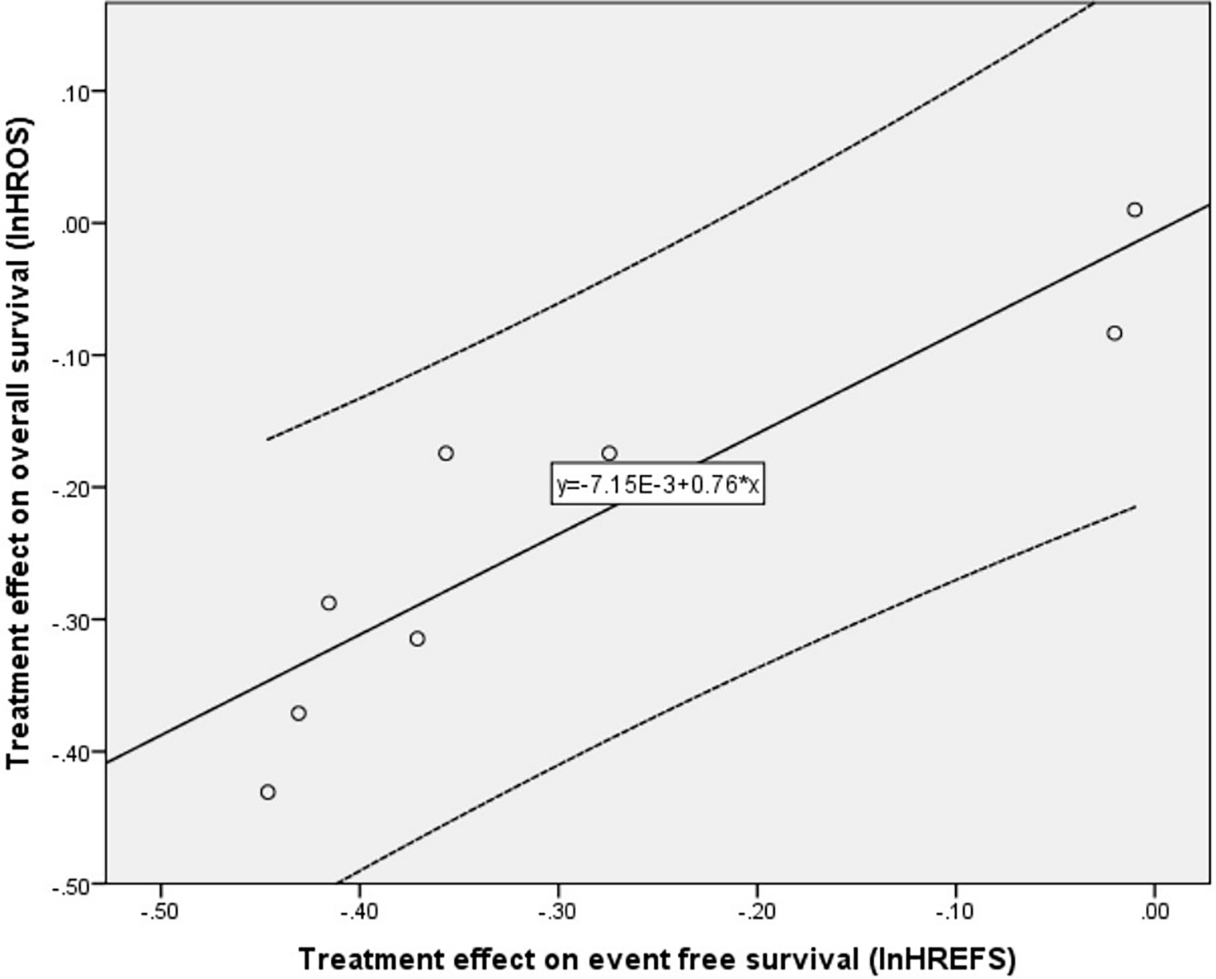
The linear regression correlation between HRs of OS and EFS and observed and predicted HRs of OS had 95% prediction limitations. Small circles: the observed effect. Black dotted line: 95% limitation of prediction. Solid black line: the predicted effect. HR, hazard ratio; OS, overall survival; EFS, event-free survival.

The data analyses of correlation here were all based on the trial level. The line regression model was lnHR_OS_ = 0.76 * lnHR_EFS_ − 7.15E−3 (R^2^ = 0.826, adjusted R^2^ = 0.797, p<0.01, **Figure 3**). There was a strong linear association between the treatment effect on OS and EFS at the trial level.

### Publication Bias Assessment

**Figure 4:**
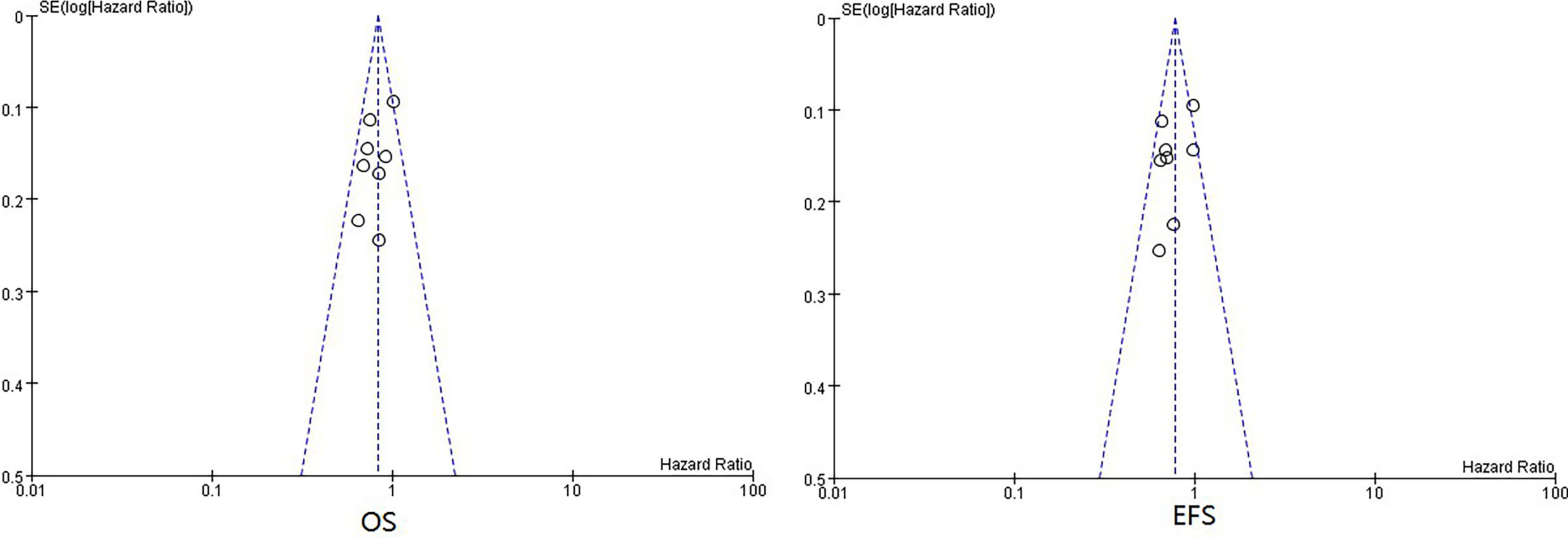
Funnel plots of HRs of EFS and OS from all included neoadjuvant trials. HR, hazard ratio; EFS, event-free survival; OS, overall survival.

There was no obvious publication bias according to the summary of bias evaluation of funnel plots (**Figure 4**).

## Discussion

Although OS is the standard primary endpoint of clinical trials for cancer treatment, a long follow-up period and a rough calculation of death of any cause stimulate the attempt to determine a suitable surrogate endpoint for OS. Studies have been developed to clarify the possibility, and results were obtained. Koji Oba et al. (17) selected 14 RCTs of adjuvant chemotherapy vs. surgery alone and collected individual survival data. The HR_OS_ and HR_DFS_ of each trial were pooled, and the correlation for two endpoints was assessed at the individual level and trial level. Spearman’s and regression analyses showed a strong correlation in both aspects (rs = 0.974, 95% CI 0971–0.976; adjusted R^2^ = 1.000, 95% CI 0.999–1.000). Extra validation of studies revealed that the predicted HR_OS_ from the observed HR_DFS_ was near the observed HR_OS_ and within the 95% prediction CI. Therefore, the therapeutic effect on DFS could be a reasonable prediction of that on OS, and DFS can be used as the primary endpoint in future clinical trials of adjuvant chemotherapies. However, the exploration of the relative possibility for trials of NCT or NCRT was unfavorable. Two analyses evaluated whether DFS could do so on GC; however, totally opposite outcomes were reached. Petrelli et al. (18) analyzed 22 neoadjuvant trials of gastroesophageal cancers to assess the effect association between pathological complete response (pCR) or DFS and OS. ADK and squamous cell carcinomas of the stomach, esophagus, or GEJ were all included. The analysis showed that median DFS and OS had a good link at the individual level (R^2^ = 0.89), while log(HR_OS_) and log(HR_DFS_) at the trial level had poor links from the above 16 studies (R^2^ = 0.27). It concluded that OS could not be surrogated in terms of NCT or NCRT effect on GC, GEJ, and esophageal cancer, although the association between two endpoints of GC could be better than esophageal cancer (R^2^ = 0.78 vs. 0.2). Ronellenfitsch et al. (19) drew an inconsistent conclusion. HR_OS_ and HR_DFS_ were calculated for each trial using individual data relying on the Kaplan–Meier method and then pooled for a combined estimation effect by meta-analyses separately. Meta-analysis showed that the estimated effect of NCT on DFS was similar to that of surgery alone (HR_DFS_: 0.86, 95% CI 0.75–0.99; HR_OS_: 0.87, 95% CI 0.75–0.99). A strong association between two endpoints was observed at both the individual level (rs = 0.8943) and trial level (R^2^ = 0.912, 95% CI: 0.75–1.0). The provided surrogate threshold effect of DFS for OS was 0.79. Based on the above statistical results, researchers concluded that DFS could be a suitable surrogate endpoint for OS in NCT or NCRT trials. However, the tumor histology and location of the study population enrolled were not limited to GC or GEJ ADK. Squamous cell carcinoma, mixed adenosquamous carcinoma, or ADK from the thoracic esophagus was not excluded. The selection process of the endpoint should be careful about hypotheses to be proven (1). The interpretation of the final results and conclusions needs much more caution.

In terms of our exploration, although the study by Shapiro et al. included enrolled ADK and squamous cell carcinoma patients, HRs of the ADK subgroup were available and included in our final analysis. Two studies with a small part of the population diagnosed with ADK in the lower third esophagus were included considering lower esophageal ADK close to GC ADK in histology. Most esophageal cancers are tumors of squamous cell carcinoma. ADK of the lower esophagus is quite similar to even distal GC, showing that esophageal ADK relies more on GC than on esophageal cancers. Similarly, robust molecules also strongly demonstrated that GEJ cancers are closer to GC than esophageal cancers, regardless of heterogeneity (20). Our study pooled HR_OS_ and HR_EFS_ provided by trials, and the pooled results were similar, displaying a superior effect of therapeutic strategy in the experimental arm (HR_OS_: 0.83, HR_EFS_: 0.78). Correlation analysis revealed a good association between the two endpoints (R^2^ = 0.826). The results and conclusions were consistent with those of Ronellenfitsch et al. (19). According to the review, when data at the individual level were not available, surrogate endpoints could be applied in trial-level decisions, although they may not be used for individual-level decisions (21). Thus, despite the lack of individual-level data in our study, EFS as a surrogate endpoint could be feasible for neoadjuvant randomized clinical trials.

Our study has limitations. First, two included studies enrolled patients with lower third esophageal ADK, which may introduce bias. Then, most included trials were conducted in European countries and parts of Korea and Japan. Evidence is deficient for other countries and regions. The above limitations restrict the wide application of conclusions. Additionally, due to potential differences in the definition of EES/PFS/DFS and assessment criteria for progression and recurrence in clinical trials, the application of conclusions in actual trials should be more cautious. Our study is the first to offer proof of the probability of EFS as a surrogate of OS in NCT or NCRT clinical trials of GC or GEJ ADK. Further analyses of individual data and trials from other regions are expected to provide more evidence.

In summary, a strong correlation was observed between OS and EFS at the trial level. EFS could be a surrogate endpoint for neoadjuvant RCTs of GC and GEJ adenocarcinoma. Further studies and evidence from individual data are expected.

## Data Availability Statement

The original contributions presented in the study are included in the article. Further inquiries can be directed to the corresponding author.

## Author Contributions

HL: conceptualization, investigation, methodology, data curation, formal analysis, and writing—original draft. YW, CQ, and TX: data curation, formal analysis, and supervision. ZP, JL, and LS: conceptualization, investigation, methodology, and supervision. XZ: conceptualization, investigation, methodology, data curation, supervision, and project administration. All authors contributed to and approved the final revision submitted.

## Conflict of Interest

The authors declare that the research was conducted in the absence of any commercial or financial relationships that could be construed as a potential conflict of interest.

The reviewer YW declared a shared affiliation with the authors to the handling editor at the time of the review.

## Publisher’s Note

All claims expressed in this article are solely those of the authors and do not necessarily represent those of their affiliated organizations, or those of the publisher, the editors and the reviewers. Any product that may be evaluated in this article, or claim that may be made by its manufacturer, is not guaranteed or endorsed by the publisher.
